# Characterization of a novel thermophilic cyanobacterium within *Trichocoleusaceae*, *Trichothermofontia sichuanensis* gen. et sp. nov., and its CO_2_-concentrating mechanism

**DOI:** 10.3389/fmicb.2023.1111809

**Published:** 2023-04-27

**Authors:** Jie Tang, Huizhen Zhou, Ying Jiang, Dan Yao, Krzysztof F. Waleron, Lian-Ming Du, Maurycy Daroch

**Affiliations:** ^1^School of Food and Bioengineering, Chengdu University, Chengdu, Sichuan, China; ^2^School of Environment and Energy, Peking University Shenzhen Graduate School, Shenzhen, China; ^3^Department of Pharmaceutical Microbiology, Faculty of Pharmacy Medical University of Gdańsk, Gdańsk, Poland

**Keywords:** 16S rRNA, 16S-23S ITS, CO2-concentrating mechanism, thermophilic cyanobacterium, *Trichocoleusaceae*, *Leptolyngbyaceae*, *Pinocchia*, *Trichothermofontia*

## Abstract

Thermophiles from extreme thermal environments have shown tremendous potential regarding ecological and biotechnological applications. Nevertheless, thermophilic cyanobacteria remain largely untapped and are rarely characterized. Herein, a polyphasic approach was used to characterize a thermophilic strain, PKUAC-SCTB231 (hereafter B231), isolated from a hot spring (pH 6.62, 55.5°C) in Zhonggu village, China. The analyses of 16S rRNA phylogeny, secondary structures of 16S-23S ITS and morphology strongly supported strain B231 as a novel genus within *Trichocoleusaceae*. Phylogenomic inference and three genome-based indices further verified the genus delineation. Based on the botanical code, the isolate is herein delineated as *Trichothermofontia sichuanensis* gen. et sp. nov., a genus closely related to a validly described genus *Trichocoleus*. In addition, our results suggest that *Pinocchia* currently classified to belong to the family *Leptolyngbyaceae* may require revision and assignment to the family *Trichocoleusaceae*. Furthermore, the complete genome of *Trichothermofontia* B231 facilitated the elucidation of the genetic basis regarding genes related to its carbon-concentrating mechanism (CCM). The strain belongs to β-cyanobacteria according to its β-carboxysome shell protein and 1B form of Ribulose bisphosphate Carboxylase-Oxygenase (RubisCO). Compared to other thermophilic strains, strain B231contains a relatively low diversity of bicarbonate transporters (only BicA for HCO_3_^−^ transport) but a higher abundance of different types of carbonic anhydrase (CA), β-CA (*ccaA*) and γ-CA (*ccmM*). The BCT1 transporter consistently possessed by freshwater cyanobacteria was absent in strain B231. Similar situation was occasionally observed in freshwater thermal *Thermoleptolyngbya* and *Thermosynechococcus* strains. Moreover, strain B231 shows a similar composition of carboxysome shell proteins (*ccmK1-4*, *ccmL*, -*M*, -*N*, -*O*, and -*P*) to mesophilic cyanobacteria, the diversity of which was higher than many thermophilic strains lacking at least one of the four *ccmK* genes. The genomic distribution of CCM-related genes suggests that the expression of some components is regulated as an operon and others in an independently controlled satellite locus. The current study also offers fundamental information for future taxogenomics, ecogenomics and geogenomic studies on distribution and significance of thermophilic cyanobacteria in the global ecosystem.

## Introduction

Thermophilic cyanobacteria are widely distributed in hot spring ecological niches (Miller et al., 2007; Alcorta et al., 2020). Moreover, many studies have demonstrated the importance of thermophilic cyanobacteria as primary photosynthetic producers of geothermal ecosystems, accounting for a large part of those ecosystems’ biomass and productivity (Esteves-Ferreira et al., 2018; Chen et al., 2021). Besides, high-value-added products can be harvested from thermophilic cyanobacteria and have been applied to numerous industries, e.g., agricultural, pharmaceutical and nutraceutical (Patel et al., 2019).

Isolation of thermophilic cyanobacteria from diverse ecosystems is critical for multidisciplinary studies regarding their morphology, genetics, physiology, biochemistry, and for providing potential strains for biotechnology and industrial applications (Cordeiro et al., 2020). It is, however, challenging to assign the taxonomy of thermophilic cyanobacteria due to their simple morphology. As a result, the diversity of thermophilic cyanobacterial genera might be severely underestimated, and community efforts are being made to rectify it. *Synechococcus*-like strains (Tang et al., 2022c) and *Leptolyngbya*-like strains (Mai et al., 2018; Yao et al., 2021) have undergone an extensive reevaluation based on multi-locus sequence analysis and genomic data, providing new insights into genetic diversity of the two genera. Furthermore, polyphasic taxonomic classification approaches have been widely utilized for cyanobacterial identification, particularly for understudied or unresolved polyphyletic families/genera/species and identification of novel families and genera (Raabova et al., 2019; Shalygin et al., 2020). Consequently, these reassignments will better taxonomically resolve cyanobacterial genera and families. Especially, the increased number of genomic sequences will facilitate the development of taxogenomics and complement the traditional 16S rRNA-based taxa identification.

The aquatic environments where thermophilic cyanobacteria live are characterized by low availability of CO_2_, primarily due to external factors, e.g., temperature, pH and gas exchange (Durall and Lindblad, 2015). Meanwhile, the availability of dissolved inorganic carbon in form of carbonates is highly variable. Therefore, to survive in the hostile aquatic habitat, the thermophilic cyanobacteria utilize CO_2_-concentrating mechanisms (CCM) to ensure that the Ribulose bisphosphate Carboxylase-Oxygenase (RubisCO) with low affinity for CO_2_ is surrounded by high CO_2_ levels and functions regardless of thermal stress (Galmés et al., 2015, 2016). Therefore, investigating the molecular component at the genomic level is a prerequisite for understanding the thermophilic cyanobacterial CCM and its relationship with their niches. In addition, under the current scenario of global warming, the studies on the molecular components of cyanobacterial CCM in relation to their specific habitats may shed light on the evolution of hot spring genomes as an example of selective pressure in warmer environments.

In our previous study, we isolated a *Leptolyngbya*-like strain, B231, from a green microbial mat of a hot spring in Zhonggu village, Sichuan, China, which can grow at 47°C and/or at the concentration of 0.1 M NaHCO_3_ (Tang et al., 2018a). Herein, thorough polyphasic characterization for strain B231, including 16S rRNA phylogeny, the secondary structure of 16S-23S ITS, and morphology description has been achieved. According to the botanical code, a new genus name *Trichothermofontia sichuanensis* gen. et sp. nov. has been proposed for strain B231 as the first representative of the genus *Trichothermofontia*. Moreover, based on our research interests, the molecular basis of CCM has been investigated for strain B231 by computational identification, and the CCM component has been further related to its adaptation. The current study lays a foundation for future taxogenomic, ecogenomic and geogenomic studies on the distribution and significance of thermophilic cyanobacteria.

## Materials and methods

### Origins, cultivation, and basic physiological assessment of *Trichothermofontia sichuanensis* B231

The strain B231, capable of forming mats, used in the present study was initially isolated from a hot spring around Zhonggu village in Ganzi Prefecture of Sichuan Province, China. Sample collection was done on 12 May 2016, with the humidity close to 71%. The ambient temperature at the time of collection was 15°C, and the light intensity was around 1,000 lux. The temperature of the hot spring, its pH, and the concentration of total dissolved solids were 55.5°C, 6.62, and 492 mmol L^−1^, respectively. Information about the sampling site and preliminary taxonomic assignment of the strain was detailed in our previous studies (Tang et al., 2018a,b). A unicyanobacterial culture of B231, recovered from 10% DMSO stocks maintained at −80°C for over 2 years, was used to establish experimental cultures as described previously (Tang et al., 2018a). Briefly, the recovered strain was cultivated at 45°C in 150 mL BG-11 medium in 500 mL Erlenmeyer flasks agitated at 100 rpm under 16 l:8D photoperiod at 45 μmol m^−2^ s^−1^ provided by fluorescent tubes unless stated otherwise. The strain initially denoted and maintained in Peking University Algae Collection as PKUAC-SCTB231 has also been deposited in the Freshwater Algae Culture Collection at the Institute of Hydrobiology (FACHB-collection) with accession number FACHB-3573. The strain was assessed for the capacity to utilize nitrite and nitrate using the modifications of BG-11, essentially as described earlier (Liang et al., 2019). Briefly, BG-11 medium without nitrogen was supplemented with 0, 0.075, 0.5, 1.5, 5, 5.7 g L^−1^ NaNO_3_; 1.218 g L^−1^ NaNO_2_. The effect of bicarbonate has been tested in regular BG-11 medium supplemented with 0, 0.1, 0.3, 0.5, 0.7, 1.0 M sodium bicarbonate. The growth of the strain has been assessed qualitatively due to the mat-forming character of the strain.

### Genome sequencing, assembly, and annotation

Integrated sequencing strategies employing MGISEQ-2000 PE150 and Oxford Nanopore Technologies PromethION sequencing platforms were applied for whole-genome sequencing of the strain B231. The sequencing was performed by a commercial provider BGI-TECH (Wuhan, China). The genomic DNA was isolated using FastDNA™ SPIN Kit for Soil (MP Biomedicals, Irvine, CA, United States), and its integrity verified with agarose electrophoresis. After sequencing, a total of 806,280,450 bp of short read data and 1,717,621,070 bp of the long read data of an average length of 14,717 bp were used for the assembly of the genome yielding a single circular chromosome and no plasmids. The assembly has been performed from long-read data using Flye v2.7 (Kolmogorov et al., 2019) module integrated into the commercial Geneious Prime 2022.2 package (Kearse et al., 2012) and subsequently refined with short-read data using Geneious mapper on default settings. The genome of strain B231 was annotated using a modified pipeline previously established by Tang et al. (2019). Briefly, gene prediction and annotation were automatically performed using the NCBI prokaryotic genome annotation pipeline (O'Leary et al., 2016), and further using the RAST annotation system (Brettin et al., 2015) to minimize poor calls. The genome annotation of strain B231 was summarized in Supplementary Table 1. The complete genome has been deposited in GenBank under accession number CP110848.

### Phylogenetic inference of 16S rRNA

The full-length 16S rRNA gene sequence of strain B231 was extracted from its genome sequence. Additional 97 16S rRNA gene sequences of cyanobacterial references were retrieved from NCBI through BLASTN search. Muscle complemented in Mega7 (Kumar et al., 2016) was employed to generate multiple sequence alignments, and manual editing comprising trimming to the same length and adjusting poorly aligned regions were carried out where necessary. Sequences of the alignment were trimmed to the same length (1,013 bp). The alignment of 16S rRNA gene sequences was subjected to Bayesian Inference using MrBayes v3.2.7 (Ronquist et al., 2012). The following parameters were applied: NST = 6, Rates = equal, MCMC Ngen = 10,000,000. Default settings were used for all the other parameters. The Bayesian analysis had a mean estimated sample size (ESS) exceeding 4,700 for all parameters, far above the average of 200 typically accepted as sufficient by phylogeneticists (Drummond et al., 2006). The final average standard deviation of split frequencies was 0.002624. The potential scale reduction factor (PSRF) value for all the estimated parameters in the Bayesian analysis was 1.00, indicating that convergence of the MCMC chains was statistically achieved (Gelman and Rubin, 1992).

### Analysis of 16S-23S ITS

The conserved domains of the 16S-23S ITS region: D1-D1’, D2, D3, boxA, and D4; and its variable regions (V2, boxB, and V3) were identified as previously described (Iteman et al., 2000). The tRNAs presented in the spacer were identified by tRNAscan-SE v1.3.1 (Lowe and Eddy, 1997). The secondary structures of the identified fragments were individually determined by Mfold web server (Zuker, 2003). Except for using the structure, draw mode untangle with loop fix, default conditions in Mfold were used in all cases.

### Whole-genome comparisons

A genome dataset was compiled for whole-genome comparisons, including genomes of strain B231 and 17 representative focus taxa (one representative from the family *Trichocoleusaceae*, eight from the family *Oculatellaceae* and nine from the family *Leptolyngbyaceae* as references). The quality of each genome was assessed using CheckM (Parks et al., 2015) to ensure a high-quality dataset with near completeness (≥95%) and low contamination (<5%). Moreover, the corresponding protein sequences of each genome were downloaded from the NCBI database.

Three indices useful for genus delineation were calculated to summarize the similarity or distance between genomes. The whole genome average nucleotide identity (ANI) and average amino acid identity (AAI) between genomes were calculated using the ANI/AAI calculator with default settings.^1^ The percentages of conserved proteins (POCP) between genomes were determined according to the method described previously (Qin et al., 2014).

### Phylogenomic reconstruction

The phylogenomic relationship between strain B231 and focal taxa was elucidated by the concatenated protein sequences from 647 single-copy genes shared by all the genomes. The homologous gene clusters identified by OrthoMCL (Li et al., 2003) were used to refine single-copy genes shared by all the genomes, which were concatenated employing a custom Perl script. Multisequence alignment was performed using MAFFT v7.453 (Standley, 2013). The supergene alignment was subjected to phylogenomic inference using IQ-TREE v2.1.3 (Minh et al., 2020). A total of 546 protein models were used to select the optimal substitution model for phylogenomic analysis using ModelFinder implemented in IQ-TREE. Bootstrap tests (1,000 replicates) were carried out for the assessment of tree topologies using UltraFast Bootstrap (Hoang et al., 2018).

### Investigation on CCM in strain B231

Orthologous proteins involved in CCM of strain B231 were identified as previously described (Tang et al., 2022d). Briefly, amino acid sequences of 28 proteins involved in CCM of *Synechocystis* sp. PCC 6803 were retrieved as a reference protein set. Orthologous genes in strain B231 were identified with the bidirectional best hit (BBH) criterion using BLASTP with the following thresholds: *E*-value cut-off of 1E-6, ≥30% identity and 70% coverage, and manually curated. Amino acid sequences of RubisCO large subunit (*rbcL*) were collected for strain B231 and reference cyanobacteria to infer the protein function and classification. Protein sequences of genes *ccmK*, *-L*, *-M*, *-N*, *-O*, and *-P* encoding carboxysome shell proteins were also collected for phylogenetic reconstruction. All the phylogenetic analyses were performed using Maximum-Likelihood (ML) algorithm as previously described (Tang et al., 2022c).

### Morphology investigation

All microscopic operations were performed essentially as described by Tang et al. (2021). In short, strain B231 was inspected at 400× magnification using light microscopy (LM, DP72, OLYMPUS, Japan), equipped with an image acquisition system (U-TV0.63XC, OLYMPUS, Japan). Microscopic investigations were also conducted using scanning electron microscopy (SEM; SU8100, HITACHI, Japan), and using transmission electron microscopy (TEM; HT7800, HITACHI, Japan). Cell measurements have been performed on 100 representative cells selected from six independent micrographs and presented as a range.

### Taxonomic evaluation

The classification system in this study was applied according to Komárek et al. (2014). Briefly, taxonomy assignment was determined based on phylogenetic position of the corresponding entity, as well as its morphological and ecological characters. The taxon description follows the prescriptions of the Botanical Code, International Code of Nomenclature for Algae, Fungi, and Plants (Shenzhen code; Turland et al., 2018).

## Results and discussion

### General genomic characteristics of strain B231

The complete genome of strain B231 has been achieved by integrating Oxford Nanopore and DNBSEQ sequencing systems yielding genome coverage of 387× and 181×, respectively. The B231 genome comprises a single circular chromosome with a size of 4,436,989 bp and a GC content of 53.9%. Bioinformatic annotation indicates that two ribosomal RNA (rrn) operons, 45 tRNA genes and 4,352 protein-coding sequences (CDS), were present in the B231 genome (Supplementary Table 1). The two ribosomal RNA operons differed in length by an 11 bp insertion in the ITS region, two single bp insertions in the 23S rRNA gene region and 2 bp difference between the two variants of 16S rRNA gene, indicating their 99.98% identity. None of the above-mentioned differences fundamentally impacted the phylograms or predicted secondary structures of the ITS region, as the 11 bp insertion was outside the regions of interest. Approximately 48.2% of protein-coding genes (2,098 out of 4,352) protein-coding genes were predicted to be hypothetical proteins. It was not surprising to identify such a high percentage of hypothetical protein in the B231 genome since similar observations are typical in the genomes of other thermophilic cyanobacteria (Cheng et al., 2020; Kono et al., 2022; Tang et al., 2022a,b).

### Phylogeny reconstruction of 16S rRNA gene

**Figure 1:**
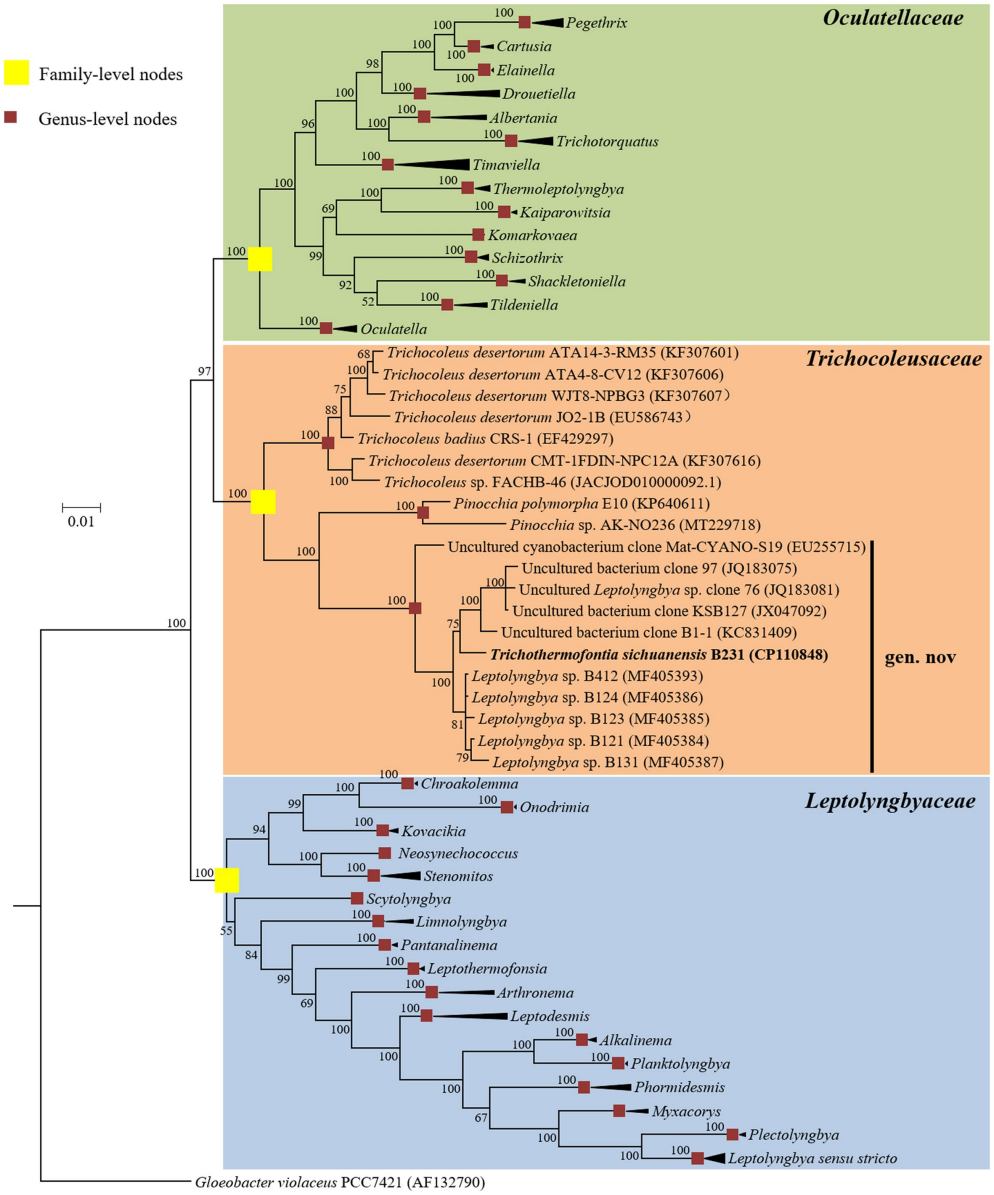
Bayesian inference of 16S rRNA gene sequences representing 98 cyanobacterial strains. Collapsed genera are indicated by black polygons, with a length corresponding to the distance from the most basal sequence to the most diverged sequence of the genus. The complete phylogram refers to Supplementary Figure 1. Posterior probabilities (%) are given above the nodes.

The Bayesian phylograms (Figure 1; Supplementary Figure 1) inferred by 16S rRNA gene sequences categorize the cyanobacterial strains into three well-defined families. Genera were also taxonomically resolved within families with the support of high posterior probabilities. The focal strain B231 is closely grouped with five thermophilic strains isolated from various hot springs in Zhonggu village (Table 1). Moreover, five other uncultured cyanobacteria were also closely related to strain B231. These 11 strains formed a well-supported clade distinct from the two described genera within the family *Trichocoleusaceae*. This result suggests the phylogenetically novel clade and a new genus within the family *Trichocoleusaceae*. In fact, the family *Trichocoleusaceae* is a monophyletic family that has been recently proposed by dividing the *Leptolyngbyaceae* into family-level clades based on molecularly-supported data (Mai et al., 2018). To date, the genus *Trichocoleus* (Mühlsteinova et al., 2014) was the only described member of the family *Trichocoleusaceae*. The phylogram generated in this study suggests the existence of two more genera in this family. The genus *Pinocchia* (Dvorak et al., 2015) is still classified within the family *Leptolyngbyaceae*, which contradicts the 16S-based phylogeny obtained in this study. Therefore, we propose that the genus *Pinocchia* should be reclassified into the family *Trichocoleusaceae*.

**Table 1:** The sequence identities of 16S rRNA gene between strain B231 and other closely related strains.

Strain	Sequence identity with B231 (%)	Isolation source	Temperature and pH	References
*Trichothermofontia sichuanensis* B231	100 (1,501)	Hot spring in Zhonggu village, Ganzi Prefecture, China, green microbial mat	55.5°C, 6.62	Tang et al. (2018b)
*Leptolyngbya* sp. B131	99.2 (1,355)	Hot spring in Zhonggu village, Ganzi Prefecture, China, green microbial mat, 3 m from B231 spring	53.1°C, 6.35	Tang et al. (2018b)
*Leptolyngbya* sp. B121	98.8 (1,355)	Hot spring in Zhonggu village, Ganzi Prefecture, China, green microbial mat, 3 m from B231 spring	53.1°C, 6.35	Tang et al. (2018b)
*Leptolyngbya* sp. B124	98.8 (1,355)	Hot spring in Zhonggu village, Ganzi Prefecture, China, green microbial mat, 3 m from B231 spring	53.1°C, 6.35	Tang et al. (2018b)
*Leptolyngbya* sp. B412	98.8 (1,355)	Hot spring in Zhonggu village, Ganzi Prefecture, China, sediment from cooler external part of the spring, 500 m from B231 spring	85.0°C, 8.50	Tang et al. (2018b)
*Leptolyngbya* sp. B123	98.7 (1,355)	Hot spring in Zhonggu village, Ganzi Prefecture, China, green microbial mat, 3 m from B231 spring	53.1°C, 6.35	Tang et al. (2018b)
Uncultured bacterium clone KSB127	98.0 (1,457)	Marine hot spring, Kalianda Island, Indonesia	NA	NA
Uncultured *Leptolyngbya* sp. clone 76	97.9 (1,453)	Marine hot spring, Kalianda Island, Indonesia	NA	NA
Uncultured bacterium clone 97	97.8 (1,457)	Marine hot spring, Kalianda Island, Indonesia	NA	NA
Uncultured bacterium clone B1-1	97.6 (1,455)	Sediment in Betong hot spring, Yala province, Thailand	NA	NA
Uncultured cyanobacterium clone Mat-CYANO-S19	96.7 (1,416)	Freshwater mesophilic microbial mat, United States	NA	NA

The number in brackets indicates the pairwise alignment length. Strains are sorted by order of identity from high to low. NA, not available.

Furthermore, the helices within 16S rRNA have been studied. Only helices 18, 23 and 27 were investigated as they were considered the most informative for family distinction (Mai et al., 2018). As shown in Table 2, one distinctive nucleotide was found in helix 18 between *Trichocoleusaceae* and *Oculatellaceae*/*Leptolyngbyaceae*. Within helix 23, two types of distinctive nucleotides were present in *Trichocoleusaceae*, both capable of differentiating them from *Oculatellaceae* and *Leptolyngbyaceae*. However, the distinctive nucleotides in helix 27 were shared among families. Taken together, several nucleotide positions in different helices were considered informative indicators of family-level classification. Again, these results confirm the recognition of the novel clade and *Pinocchia* to join *Trichocoleus* as members of the family *Trichocoleusaceae*.

**Table 2:** Nucleotide comparisons of focal helices within 16S rRNA among families.

Family	Helix 18	Helix 23	Helix 27
*Trichocoleusaceae*	TGCCAGCAGCCGCGGTAA**G**A	ATCGGGAAGAACACC**A**G**T**G ATCGGGAAGAACACC**A**G**A**G (*Pinocchia*)	GGGAGTACGC**T**CGCAAG**A**GTGAAACTC GGGAGTACGC**A**CGCAAG**T**GTGAAACTC (*Pinocchia*)
*Oculatellaceae*	TGCCAGCAGCCGCGGTAA**T**A	ATTRGRAAGAACAYC**G**G**T**G	GGGAGTACGC**T**CGCAAG**A**GTGAAACTC
*Leptolyngbyaceae*	TGCCAGCAGCCGCGGTAA**T**A	ATTGGGAAGAACACC**A**G**C**G	GGGAGTAYGC**A**CGCAAG**T**GTGAAACTC

Nucleotides variable between families but consistent within families are in bold. IUPAC code letters are assigned for nucleotides that vary within the consensus sequences.

Within the novel clade, all the strains show high 16S rRNA sequence identities (97.6%–99.2%) to the strain B231 except for clone Mat-CYANO-S19 (Table 1). Therefore, according to the recommended threshold of 16S rRNA gene identity for bacterial species (98%–99%) or genera (94.5%–95%) demarcation (Yarza et al., 2014), clone Mat-CYANO-S19 can be proposed to be new species and the remaining 10 strains to be another new species. Such delineation was also supported by the 16S rRNA phylogeny (Figure 1). Besides, the novel clade shows 92.8%–93.5% of 16S rRNA sequence identities to *Trichocoleus* strains and 91.3%–92.9% to *Pinocchia* strains. This result was consistent with the taxonomic delineation of this novel clade as a new genus member within the family *Trichocoleusaceae*.

Intriguingly, the habitat niches were quite distinct among strains from the novel clade (Table 1). The six strains isolated from China were originally recovered from three separate freshwater hot springs located at a high altitude (3,200 m; Tang et al., 2018a,b). The other five strains, for which only their molecular signatures were available, also mostly show thermal origin. Three were from marine hot springs in Indonesia, one from a mesophilic microbial mat in the United States and one from a hot spring sediment in Thailand. The distinct habitat niches suggested that these strains might be classified into at least three ecotypes. The ecotype determination of uncultured bacterium clone B1-1 appears impractical in light of the minimal ecological information. In addition, the acclimation of these strains to diverse habitats strongly suggests the underlying genetic diversity and a wide distribution among representatives of this genus. Verifying this speculation will be an interesting topic that could be explored using phylogenomic and ecogenomic approaches providing that more isolates and genome sequences are available. Unfortunately, the data on uncultured strains were restricted to molecular signatures isolated from environments, hindering further detailed comparisons (e.g., physiological, ecological and genomic studies) that would require isolates.

### Secondary structures of 16S-23S ITS

In the present study, the phylogeny of 16S-23S ITS has not been reconstructed mainly for two reasons. First, the 16S-23S ITS of important reference cyanobacteria, *Pinocchia* strains, only contained conserved tRNA^lle^, while another conserved tRNA^Ala^ was missing (Dvorak et al., 2015). Second, the 16S-23S ITS of strain B231 lacks the V2 region (Table 3); indicating significant operon heterogeneity in these strains. Taken together, the current dataset of 16S-23S ITS sequences was extraordinarily divergent and would most likely result in a misleading taxonomic assignment (Johansen et al., 2011). However, sequence comparisons of 16S-23S ITS were still performed among representatives from the three families. Excluding two highly conserved tRNAs from ITS sequences, the length of the remaining ITS sequences tremendously varied from 230 to 535 bp (Table 3). Such an immense discrepancy was primarily attributed to the length differences in D1-D1’ (51–141 bp), V2 (0–218 bp), boxB (33–70 bp), and V3 (21–161 bp). Within family *Trichocoleusaceae*, enormous variations were also observed among genera (Table 3). As a result, the nucleotide differences of these domains result in divergences of the secondary structures among representative strains from family *Trichocoleusaceae* (Supplementary Figure 2).

**Table 3:** The Length summary (bp) of corresponding regions within 16S-23S ITS of strain B231 and focal taxa.

Family	Species	ITS length (tRNA removed)	D1-D1’ helix	D2	D3	boxA	D4	V2 helix	boxB helix	V3 helix
*Trichocoleusaceae*	*Trichothermofontia sichuanensis* B231	269	63	12	5	12	7	0	70	75
	*Trichocoleus desertorum* ATA4-8-CV12	244	62	12	5	12	7	19	37	63
	*Pinocchia polymorpha* E10	372	119	12	5	12	7	10	46	162
*Oculatellaceae*	*Albertania skiophila* SA373	320	64	12	5	12	7	46	47	22
	*Drouetiella hepatica* Uher 2000/2452	281	64	12	5	12	7	21	34	50
	*Elainella saxicola* E1	296	62	12	5	12	7	58	33	19
	*Kaiparowitsia implicata* GSE-TBC-09CA2	394	142	12	5	12	7	9	36	121
	*Komarkovaea angustata* EY01-AM2	325	64	12	5	12	7	20	41	94
	*Oculatella* sp. FACHB-28	265	64	12	5	12	7	17	34	52
	*Pegethrix bostrychoides* GSE-TBD-MK4-15B	308	87	12	5	12	7	24	36	94
	*Thermoleptolyngbya sichuanensis* A183	535	64	12	5	12	7	218	48	74
	*Tildeniella torsiva* UHER1998/13D	262	66	12	5	12	7	7	49	92
	*Timaviella obliquedivisa* GSE-PSE-MK23-08B	319	63	12	5	12	7	29	49	59
	*Trichotorquatus coquimbo* ATA2-1-KO25A	314	100	12	5	12	7	23	36	118
*Leptolyngbyaceae*	*Alkalinema pantanalense* CENA528	296	64	12	5	12	7	24	48	54
	*Chroakolemma pellucida* 719	268	61	12	5	12	7	16	41	53
	*Kovacikia muscicola* HA7619-LM3 clone 41A	345	63	12	5	12	7	90	41	95
	*Leptodesmis sichuanensis* A121	325	63	12	5	12	7	81	33	98
	*Leptolyngbya boryanum* PCC 73110	275	51	12	5	12	7	10	33	21
	*Leptothermofonsia sichuanensis* E412	380	121	12	5	12	7	76	45	98
	*Limnolyngbya circumcreta* CHAB5667	388	98	12	5	12	7	83	63	76
	*Myxacorys californica* WJT24-NPBG12B	258	86	12	5	12	7	9	33	71
	*Neosynechococcus sphagnicola* sy1	230	63	12	5	12	7	11	39	95
	*Onodrimia javanensis* 28	280	105	12	5	12	7	7	44	47
	*Phormidesmis priestleyi* ANT.L52.4	329	113	12	5	12	7	12	56	77
	*Plectolyngbya hodgsonii* ANT.LPR2.2	306	55	12	5	12	7	29	44	19
	*Scytolyngbya timoleontis* XSP2	276	64	12	5	12	7	14	40	94
	*Stenomitos rutilans* HA7619-LM2	258	65	12	5	12	7	7	34	92

The inferred D1-D1’ helix of strain B231 differs from the other two inferred structures (Supplementary Figure 2A). The most similar structure to D1-D1’ helix of strain B231 was that of *T. desertorum*. Both D1-D1’ helices vary in the overall length and topology while retaining the basal stem structures of five base pairs followed by a right asymmetrical loop.

V2 helix was absent in the 16S-23S ITS of strain B231. A similar structure of the V2 helix was shared by the other two representative strains from the family *Trichocoleusaceae*, comprising one stem and a hairpin loop (Supplementary Figure 2B). However, the V2 helices of the two strains differ in the stem length and residues of hairpins.

All three strains share a basal stem structure (AGCA-TGCT) in boxB helices (Supplementary Figure 2C). Strain B231 shows a much longer residue length than the other two strains (Table 3), resulting in a divergent boxB helix structure.

The V3 helix of strain B231 consists of an asymmetrical loop and a 4-residue hairpin loop, and two stems (Supplementary Figure 2D). The V3 helix of strain B231 was different from those of the other two strains, while a basal stem structure (TGTC-GACA) was shared by all the strains.

Conclusively, the phylogeny of 16S rRNA and the result of 16S-23S ITS secondary structure analysis supports the delineation of strain B231 as a novel genus within this family.

### Genome comparisons

To our best knowledge, there are no genomic-level comparisons within the family *Trichocoleusaceae* or between members of the family *Trichocoleusaceae* and genera from other different families. Therefore, it was crucial to elucidate a snapshot of genomic divergences between Strain B231 and focal taxa. Herein, three indices of whole genome comparisons between strain B231 and representative species from *Trichocoleusaceae*, *Oculatellaceae* and *Leptolyngbyaceae* were presented (Table 4).

**Table 4:** Indices values (%) of whole genome comparisons between strain B231 and representative species from *Trichocoleusaceae*, *Oculatellaceae* and *Leptolyngbyaceae*.

Family	Species	ANI	AAI	POCP
*Trichocoleusaceae*	*Trichocoleus desertorum* ATA4-8-CV12	71.97	61.53	47.60
*Oculatellaceae*	*Drouetiella hepatica* Uher 20,002,452	72.67	59.85	45.22
	*Elainella saxicola* E1	75.41	60.36	38.78
	*Kaiparowitsia implicata* GSE-PSE-MK54-09C	72.34	58.94	43.57
	*Oculatella* sp. FACHB-28	74.05	60.68	43.94
	*Pegethrix bostrychoides* GSE-TBD4-15B	72.83	59.77	49.01
	*Thermoleptolyngbya sichuanensis* A183	76.44	61.43	49.19
	*Tildeniella torsiva* UHER_199813D	73.45	57.84	45.65
	*Timaviella obliquedivisa* GSE-PSE-MK23-08B	71.25	59.96	49.57
*Leptolyngbyaceae*	*Alkalinema* sp. FACHB-956	74.39	58.51	49.21
	*Leptodesmis sichuanensis* A121	77.66	62.17	49.23
	*Leptolyngbya boryana* dg5	78.97	58.92	44.76
	*Leptothermofonsia sichuanensis* E412	81.51	62.07	46.79
	*Myxacorys almedinensis* A	74.17	59.78	49.23
	*Neosynechococcus sphagnicola* sy1	75.85	59.72	44.28
	*Pantanalinema* sp. GBBB05	74.03	61.23	48.76
	*Phormidesmis priestleyi* BC1401	75.61	59.87	46.91
	*Stenomitos frigidus* ULC18	72.21	59.95	45.06

Considerable divergences in genomes were observed between strain B231 and the other 18 strains, as revealed by the ANI and AAI values (Table 4). The ANI and AAI values were less than 82 and 63%, respectively. This result conforms to the suggested values for genus (ANI < 83%, AAI ≤ 70%) delimitation (Walter et al., 2017; Jain and Rodriguez, 2018). However, in some cases, misleading results might be achieved for the classification of the prokaryotic genus using ANI or AAI (Pannekoek et al., 2016). Therefore, the POCP specific for genus delineation was also calculated between strain B231 and the other focal taxa. The POCP values range from 38.78% to 49.23% (Table 4), all within the threshold (<50%) for the definition of a prokaryotic genus (Qin et al., 2014). Taken together, all the results verify the genus demarcation of strain B231 as a novel genus within the family *Trichocoleusaceae*. This conclusion was in accordance with the results of molecular phylogeny (Figure 1).

### Phylogenomic analysis

**Figure 2:**
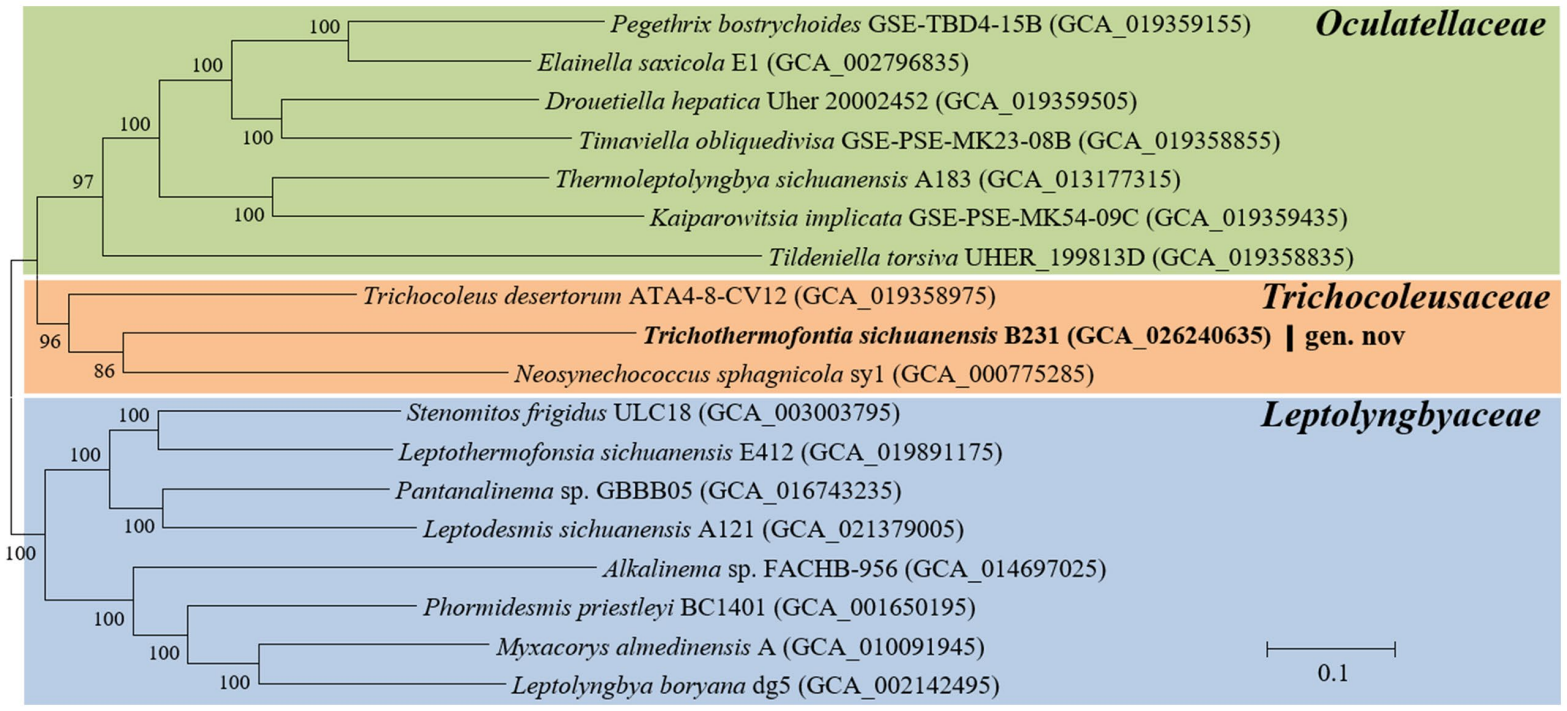
ML phylogenomic inference of concatenated protein alignment of 647 single-copy genes shared by all genomes. Strain no. in bold represent the strain identified in this study. Bootstrap values are indicated at nodes. Scale bar = 10% substitutions per site.

Analysis of homologous gene clusters generated 647 single-copy genes shared by the genomes studied. The concatenated alignments of these genes possess 216,534 aligned amino acid sites. Using the optimal substitution model (LG + F + R5), the ML inference of the supergene alignment provides a phylogeny with strong bootstrap support for all branches (Figure 2), defining representative species from each described genus. Within the *Trichocoleusaceae* clade, strain B231 is quite divergent from the described genus *Trichocoleus* and *Neosynechococcus.* Strain B231 is well-separated by the long branches from other described genera in the family, suggesting the taxon as a novel genus.

The phylogenomic topology was almost identical to that of the 16S rRNA gene (Figure 1). However, one exception was present in the phylogram. The phylogenetic analysis of the 16S rRNA gene suggests the clear affiliation of *Neosynechococcus sphagnicola* sy1 to the family *Leptolyngbyaceae* (Figure 1; Dvorak et al., 2014), whereas sy1 was located in a position within the genome-scale phylogram, forming a clade with two genera from family *Trichocoleusaceae* (Figure 2). This position of the strain was consistent with the phylogenomic results reported in previous studies (Tang et al., 2022a,b). Taken together, the discordant two trees suggest that currently used approaches cannot determine whether the taxonomic position of sy1 is accurate. The inconsistent phylogenies imply that an extensive study of this organism encompassing phenotypical, chemotaxonomical, physiological and genotypical studies should be carried out.

### Classification of CCM in strain B231

The aquatic environments where most thermophilic cyanobacteria live usually suffer from low availability of CO_2_, primarily due to external factors, e.g., temperature, pH and gas exchange. On the other hand the availability of soluble carbonates is highly variable depending on the geochemistry of the habitat. Therefore, it was crucial to investigate the molecular component and organization of CCM of thermophilic strains in relation to their habitats.

**Figure 3:**
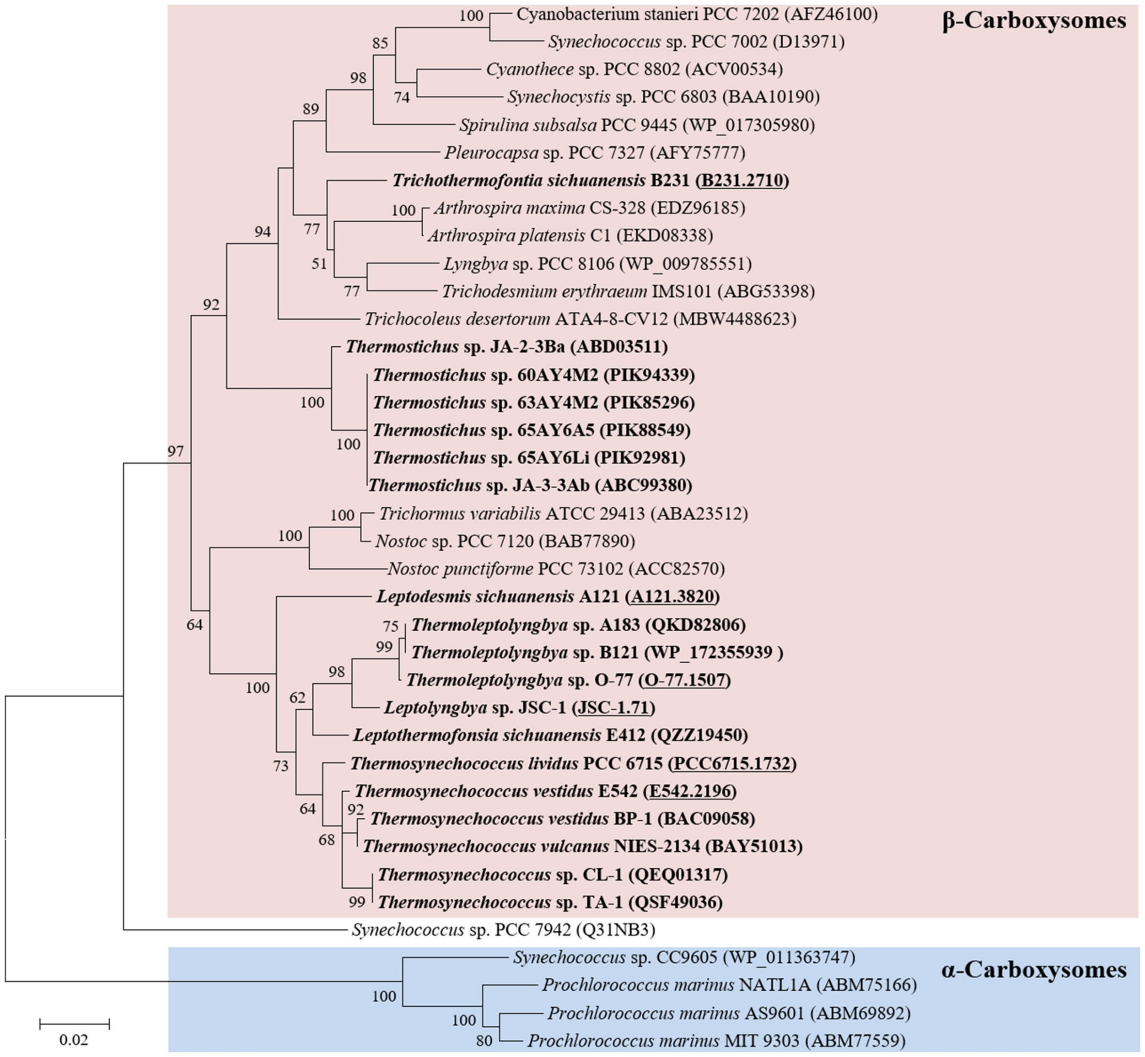
ML phylogenetic inference of RubisCO large subunit protein sequences. The thermophilic cyanobacterium investigated in this study and previously reported thermophiles are indicated in bold. The accession numbers underscored refer to the gene IDs in Supplementary Table 1 or the reference (Tang et al., 2022d).

Phylogenetic analysis of the molecular marker, *rbcL*, was frequently used to indicate the carboxysome type of cyanobacteria (Klanchui et al., 2017). Herein, the ML phylogram of *rbcL* positions strain B231 within the category of β-cyanobacteria (Figure 3), indicating the presence of RubisCO 1B form in this thermophile. This result was in accordance with a previous study that surveyed 17 thermophilic cyanobacteria and allocated them to the β-cyanobacteria (Tang et al., 2022d). Nevertheless, strain B231 clusters with none of these thermophilic cyanobacteria but is uniquely positioned among mesophilic cyanobacteria (Figure 3), suggesting considerable genetic diversity of *rbcL* amino acid sequences among these thermophiles. Furthermore, the evolutionary relationship based on habitats or morphology cannot be elucidated from the present rbcL phylogram, suggesting that the phylogenetic inference of *rbcL* may be unsuitable for elaborating the relationship between evolution and cyanobacterial habitat and environments (Komárek, 2016).

### Genes encoding Ci uptake systems in strain B231

**Figure 4:**
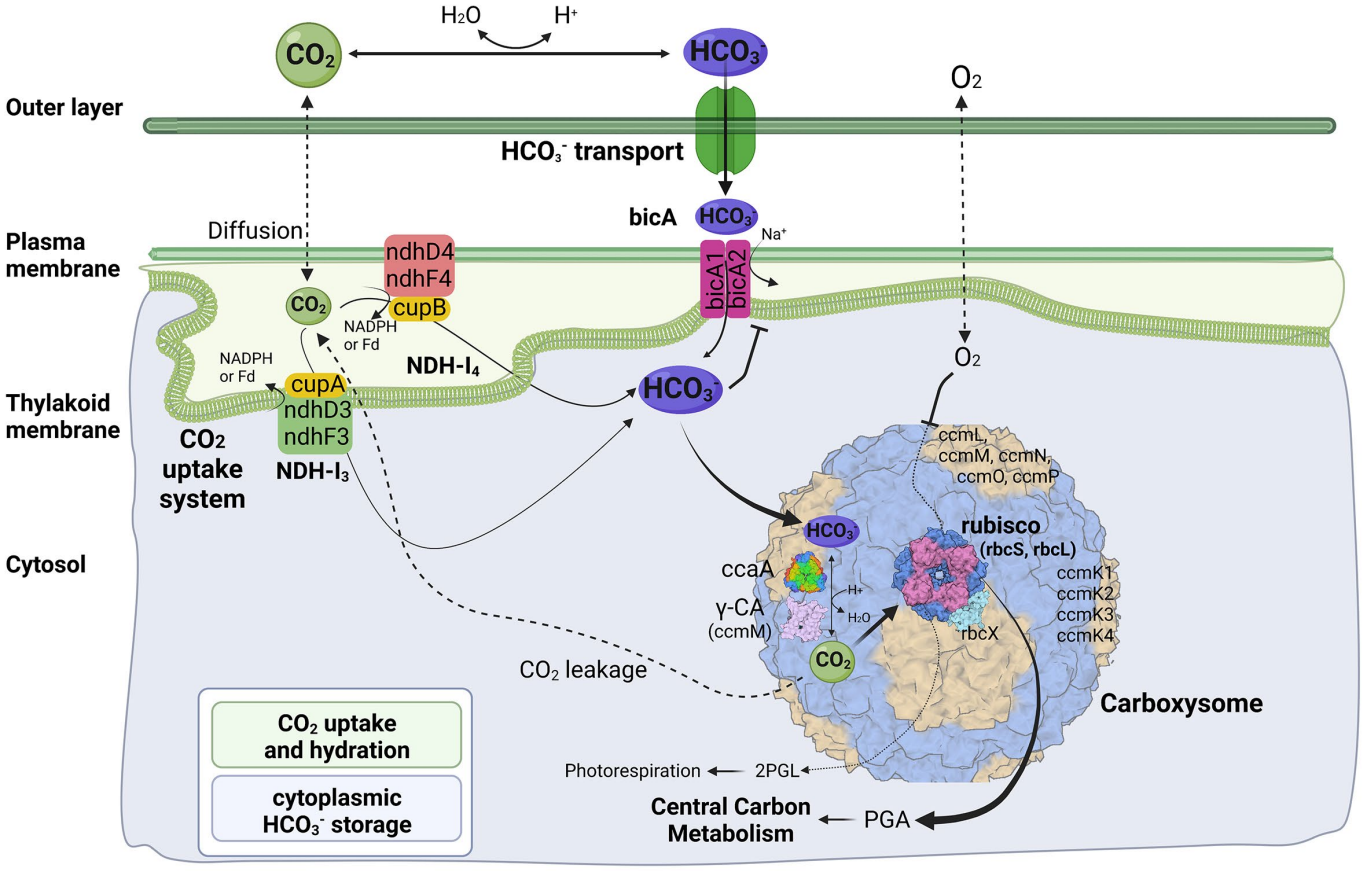
Molecular components of CCM for *Trichothermofontia sichuanensis* B231. 2PGL, 2-phosphoglycolate; PGA, phosphoglyceric acid. Three-dimensional structures of proteins visualized using related structures of 2YBV, 6OWF, 5SWC, 5BS1, 6KI1. Figure created with BioRender.com.

In general, the cyanobacterial CCM comprises two primary components: Ci uptake systems and carboxysomes. Cyanobacteria have been reported to have up to five different systems to actively acquire and transport Ci into the cells. Two for the uptake of CO_2_ and three for the transport of HCO_3_^−^ (Pronina et al., 2017). Herein, three different transport systems (Figure 4) have been identified in strain B231, including two CO_2_ uptake systems, NDH-1_3_ and NDH-1_4_ complex, and one HCO_3_^−^ transport system, BicA. Existence of both CO_2_ uptake systems in strain B231 aligns with the previous studies indicating that these CO_2_ transporters were present in β-cyanobacteria living in freshwater, brackish or eutrophic lakes (Durall and Lindblad, 2015). This is in sharp contrast to oceanic α-cyanobacteria (e.g., *Prochlorococcus* species) and marine β-cyanobacteria (e.g., *Trichodesmium* species) that contain only one or even lack them entirely (Pronina et al., 2017). Taken together, the presence of NDH-1_3_ and NDH-1_4_ might be relevant to the environments where these cyanobacteria inhabit. Indeed, a low-CO_2_ inducible high-affinity CO_2_ uptake system (NDH-1_3_ complex) and a constitutive low-affinity CO_2_ uptake system (NDH-1_4_ complex), may allow the thermophilic cyanobacteria more alternative strategies to survive in environments with significant CO_2_ fluctuation, particularly in hot springs. In addition, the protein sequences of five genes (*ndhD4*, *ndhF4*, *cupB*, *ndhD3*, *ndhF3*) encoding the two CO_2_ uptake systems exhibit different identities with the sequences of non-thermophilic reference cyanobacteria (*Synechocystis* PCC 6803), ranging from 60.4 to 68.6%, but a high degree of homology (84.4% identity) to *cupA* gene of NDH-1_3_ complex.

Two homologs of BicA were present in strain B231 (Figure 4). However, the protein sequences of the two homologs show distinct identities to that of *Synechocystis* PCC 6803, namely 66.5% and 36.7%. Such discrepancy in CDS of *bicA* genes in strain B231 requires future studies to elucidate the possible evolutionary processes and functional differences. In addition, the HCO_3_^−^ transport systems, *sbt* regulator and BCT1, were not present in strain B231. The absence of *sbt* regulator in strain B231 was consistent with the previous finding that the thermophilic cyanobacteria typically have *bicA* rather than *sbt* as suggested by their dominant presence. At the same time, a lack of BCT1 was observed in several thermophilic cyanobacteria, e.g., *Thermoleptolyngbya* and *Thermosynechococcus* strains (Tang et al., 2022d). Meanwhile, thermophiles without both *sbt* regulator and BCT1 were rare (Tang et al., 2022d). This result suggests that the BicA in strain B231 may be sufficient to manage the transport of dissolved inorganic carbon in its habitat.

### Genes encoding carboxysomes in strain B231

**Figure 5:**
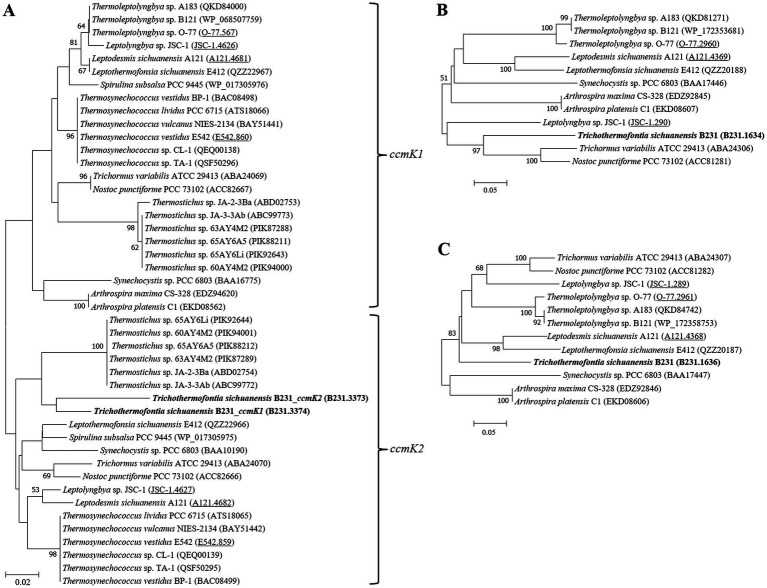
ML phylogenetic inference of protein sequences of *ccmK1/2* **(A)**, *ccmK3* **(B)**, and *ccmK4* **(C)**. The thermophilic cyanobacterium investigated in this study is indicated in bold. The accession numbers underscored refer to the gene IDs in Supplementary Table 1 or the reference (Tang et al., 2022d).

The cyanobacterial carboxysomes comprise protein shells and two encapsulated enzymes, RubisCO and carbonic anhydrase (CA; Kerfeld and Melnicki, 2016). For β-cyanobacteria, shell proteins are normally encoded by *ccmKLMNO* operon and *ccmP* (Melnicki et al., 2021). Strain B231 contains *ccmK1*-*4* (Figure 4), a typical gene set in the β-cyanobacterial genome (MacCready et al., 2020). It is known that *ccmK1* and *ccmK2* are the main structural proteins of the carboxysome shell and share high sequence conservation (Cai et al., 2016). Phylogenetic analysis suggests that the two homologs of *ccmK1/2* in strain B231 form a separate cluster, and both group into the clade of *ccmK2* (Figure 5A). Although 10 amino acid-long C-terminal extension of *ccmK1* can distinguish *ccmK1* and *ccmK2* (Kerfeld et al., 2005), the remaining part of *ccmK1/2* protein sequences share a similarity as high as 92.2%, leading to their assembly into one cluster. The structural and functional specificity of the two *ccmK* genes requires future careful investigation. The *ccmK3* and *ccmK4* of strain B231 were relatively divergent from other cyanobacteria, as suggested by long branches and substitution rates (Figures 5B,C). The *ccmK3* and *ccmK4* of strain B231 show a moderate degree of homology to that of *Synechocystis* PCC 6803, 61.5% and 74.5%, respectively. The *ccmK3/K4* present in the strain B231 may function as adjusting the properties of carboxysome for rapid adaptation to environmental changes in thermal regions through, e.g., expanding the range of permeability properties of metabolite channels in carboxysome shells (Sommer et al., 2019).

Apart from *ccmK*, other genes encoding carboxysome shell proteins, *ccmL*, -*M*, -*N*, -*O*, and -*P*, were also present in strain B231 (Figure 4). Only *ccmM* and *ccmN* show weak homologs (46.2, 32.1%) to that of *Synechocystis* PCC 6803, while the other three genes exhibit an identity of 62.2%, 61.4%, and 72.0%, respectively. Phylogenetic analysis of these genes suggests extensive genetic divergence between strain B231 and reference cyanobacteria, as suggested by the assignments of strain B231 into separate branches (Supplementary Figure 3).

The carboxysomal CA catalyzes the conversion of HCO_3_^−^ into CO_2_, a substrate for RubisCO. The subunits of RubisCO, *rbcL* and *rbcS*, and RubisCO assembly chaperone, *rbcX*, were present in strain B231 (Figure 4). The *rbcL* of strain B231 shows high sequence conservation to *Synechocystis* PCC 6803 (89.7% identity), whereas moderate conservation (66.4% identity) was observed in *rbcS*. The protein sequence of *rbcX* was more divergent, showing 58% identity with the reference protein. As for CA, only one type of β-CA, carboxysomal *ccaA* (Figure 4), was present in strain B231, with a similarity of 56.4% to the reference protein. Moreover, Strain B231 shows a similar primary structure (Supplementary Figure 4) of amino acid residues in γ-CA-like domain of *ccmM* to that with CA activity in *Thermosynechococcus* BP-1 (Peña et al., 2010) and *Nostoc* PCC 7120 (de Araujo et al., 2014), indicating that the *ccmM* protein of strain B231 may possess CA activity. Thus, the CA activities of *ccmM* and *ccaA* might confer strain B231 with more alternative strategies for regulating carboxysome function. Intriguingly, previous studies (Peña et al., 2010; de Araujo et al., 2014; Tang et al., 2022d) suggest that the CA activity of *ccmM* (γ-CA) was usually present in cyanobacteria lacking *ccaA*, while cyanobacteria comprising both *ccmM* and *ccaA* are likely to have non-functional γ-CA domain and only *ccaA* function as CA activity. Future experimental studies should be performed to elucidate the function and relationship of the two identified proteins with potential CA activity in strain B231.

### Genomic organization of CCM-related genes in strain B231

**Figure 6:**
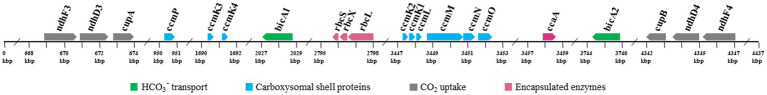
Genomic organization of CCM-related genes for *Trichothermofontia sichuanensis* B231. Solid arrow boxes refer to genes and the direction of transcription.

Investigation of the genomic organization of CCM-related genes in strain B231 may provide insights into the function and evolution of these genes. As shown in Figure 6, genes encoding each NDH-1 complex separately cluster together. The two homologs of *bicA* genes are distantly located on the genome (Figure 6). Regarding the gene organization of the carboxysomal shell proteins, a typical main carboxysome locus (MCL) was present. It contained *ccmK1/2* genes, followed by *ccmL*, -*M*, -*N*, and -*O* (Figure 6). The sequential arrangement of *ccmK2* and *ccmK1* in the MCL may facilitate protein complex assembly or balancing of the shell protein stoichiometry during the translation of MCL genes (Cai et al., 2016), while *ccmK*, -*L*, -*M*, -*N*, and -*O* may be co-regulated in an MCL as an operon (Rae et al., 2013; Billis et al., 2014; Sommer et al., 2017). In addition to the MCL, the other three shell proteins form two satellite loci, *ccmP* and an operon with *ccmK3/4* genes. The separated organization of *K1*/*K2* and *K3/K4* paralogs may be associated with the structural segregation of the two groups (Sommer et al., 2019). Moreover, the expression of *ccmK3*/*4* from a satellite locus may increase the flexibility of carboxysome shell assembly and permeability (Sommer et al., 2019), and may provide differing metabolite selectivities (Sommer et al., 2017). The localization of genes encoding *rbcL*, *rbcS* and *rbcX* for RubisCO was consistent with the previous findings that the three genes always cluster in an operon in β-cyanobacteria (Badger et al., 2002). In addition, the RubisCO gene cluster was remote from the MCL, suggesting that this gene cluster was a satellite locus to MCL and may conduct independent expression regulation. The role of *rbcX* in thermophilic cyanobacteria is still uncertain and requires future investigations.

Comparative analysis indicates that strain B231 shows a distinct molecular component to the phylogenetically closely related neighbor, *Trichocoleus* ATA4-8-CV12 (Supplementary Table 2). For the CO_2_ uptake systems, strain B231 has one set of genes encoding NDH-1_4_ complex, while *Trichocoleus* ATA4-8-CV12 has two sets of homologs. The constitutive low-affinity CO_2_ uptake system (NDH-1_4_ complex) with an extra set of genes may be indispensable for the strain to survive in its terrestrial origin (desert) where gaseous CO_2_ is the primary carbon source (Mühlsteinova et al., 2014) and bicarbonate is less available, or simply the aftermath of strain evolutionary history. More studies are needed on the topic of multiple paralogs of carbon uptake systems in cyanobacteria. Both strains have only one type of HCO_3_^−^ transport system, namely BicA in strain B231 and BCT1 in strain ATA4-8-CV12. A different type of HCO_3_^−^ transport system could be attributed to the distinct traits of the two transporters and the habitat of the two strains. First, BicA exhibits low affinity and high flux rate to HCO_3_^−^ and the genes encoding BicA were found to be primarily constitutively expressed (Price et al., 2004). Therefore, the BicA transport system might be necessary for strain B231 to acclimatize to the exogenous HCO_3_^−^ in its habitat (pH 6.62) where HCO_3_^−^ remains dominant dissolved inorganic specie between pH 6 and 10. Second, BCT1 encoded by *cmpABCD* operon shows a high affinity for HCO_3_^−^ and was induced under low levels of Ci and enhanced by high light conditions (Omata et al., 2002). Thus, strain ATA4-8-CV12 may use BCT1 to compensate for the functions of constitutive genes (e.g., BicA) for adaptation to high-light conditions in deserts. In addition, strain B231 has one type of β-CA (*ccaA*) and *ccmM* (γ-CA) with CA activity, whereas *Trichocoleus* ATA4-8-CV12 contains two types of β-CA (*ccaA* and *ecaB*) and its *ccmM* appears to have non-functional γ-CA domain (Supplementary Figure 4). The presences of other genes related to CCM are consistent with the two strains. However, the phylogenetic analysis suggests that the protein sequences are divergent (Figures 3, 5; Supplementary Figure 3). Though the genome of *Trichocoleus* ATA4-8-CV12 was only assembled as a draft, preliminary analysis suggests that its clustering pattern of CCM-related genes in the genome was in accordance with that of strain B231.

Overall, the molecular component and organization of strain B231 were similar to that of other reported thermophilic cyanobacteria (Supplementary Table 2; Tang et al., 2022d). However, distinct characteristics were evident in strain B231. A relatively low diversity of bicarbonate transporters distinguishes strain B231 from other thermophilic strains, each possessing at least two HCO_3_^−^ transport systems (Tang et al., 2022d). The low diversity may be related to the habitat of strain B231 where a higher variety of transporters with similar functionalities is not required. In addition, strain B231 contains a higher abundance of different types of carboxysomal CAs, β-CA (*ccaA*) and γ-CA (*ccmM*), compared to other thermophilic strains (β-CA or γ-CA), but still below those of other freshwater cyanobacteria that possess the abundance of CAs, α-/β-/γ-CAs (Tang et al., 2022d). The CA activities of *ccmM* and *ccaA* may provide strain B231 with alternative strategies to ensure that the RubisCO with low affinity for CO_2_ was surrounded by high CO_2_ levels and regularly function regardless of thermal stress in this habitat. Strain B231 with freshwater origin lacks the BCT1 transporter, which is near-ubiquitous in freshwater cyanobacteria. Moreover, strain B231 shows a similar composition of carboxysome shell proteins to mesophilic cyanobacteria, the diversity of which was higher than many thermophilic strains lacking at least one of the four *ccmK* genes (Tang et al., 2022d).

### Morphological and physiological features of strain B231

The primary physiological characteristics of the B231 strain were assessed by monitoring its growth at the temperature of 47°C in derivatives of BG-11 medium; the unmodified medium, supplemented with bicarbonate up to the concentration of 1.0 M, and nitrogen-free. The strain could grow in all tested nitrogen conditions, showing optimal growth with moderate (a third of the regular BG-11 medium nitrate content) to high nitrogen availability (five times the regular BG-11 medium nitrate content) and in the presence of 17.65 mM nitrite. Interestingly, despite the lack of heterocytes the strain could grow in a nitrogen-free medium, which is consistent with the presence of the *nif*HDK gene cluster and associated genes required for molecular nitrogen fixation. The strain grew optimally from ambient up to the 0.1 M bicarbonate concentration. Higher loads of dissolved inorganic carbon had a deleterious effect on the strain, concurrent with its highly restricted bicarbonate transport system revealed earlier. The results are summarized in Table 5.

**Table 5:** Physiological characteristics of *Trichothermofontia* B231 grown in 47°C, 150 μmol m^−2^ s^−1^ under varying carbonate and nitrate concentrations.

Supplemented inorganic carbon		Supplemented nitrogen	
HCO_3_^−^ (mol/L)	Growth	NO_3_^−^ (mmol/L)	Growth
0 (ambient)	++	0	+
0.1	+	0.88	+
0.3	−	5.88	++
0.5	−	17.65	++
0.7	−	58.83	++
1.0	−	88.24	++

“−” no growth; “+” poor growth; “++” baseline growth in BG-11.

**Figure 7:**
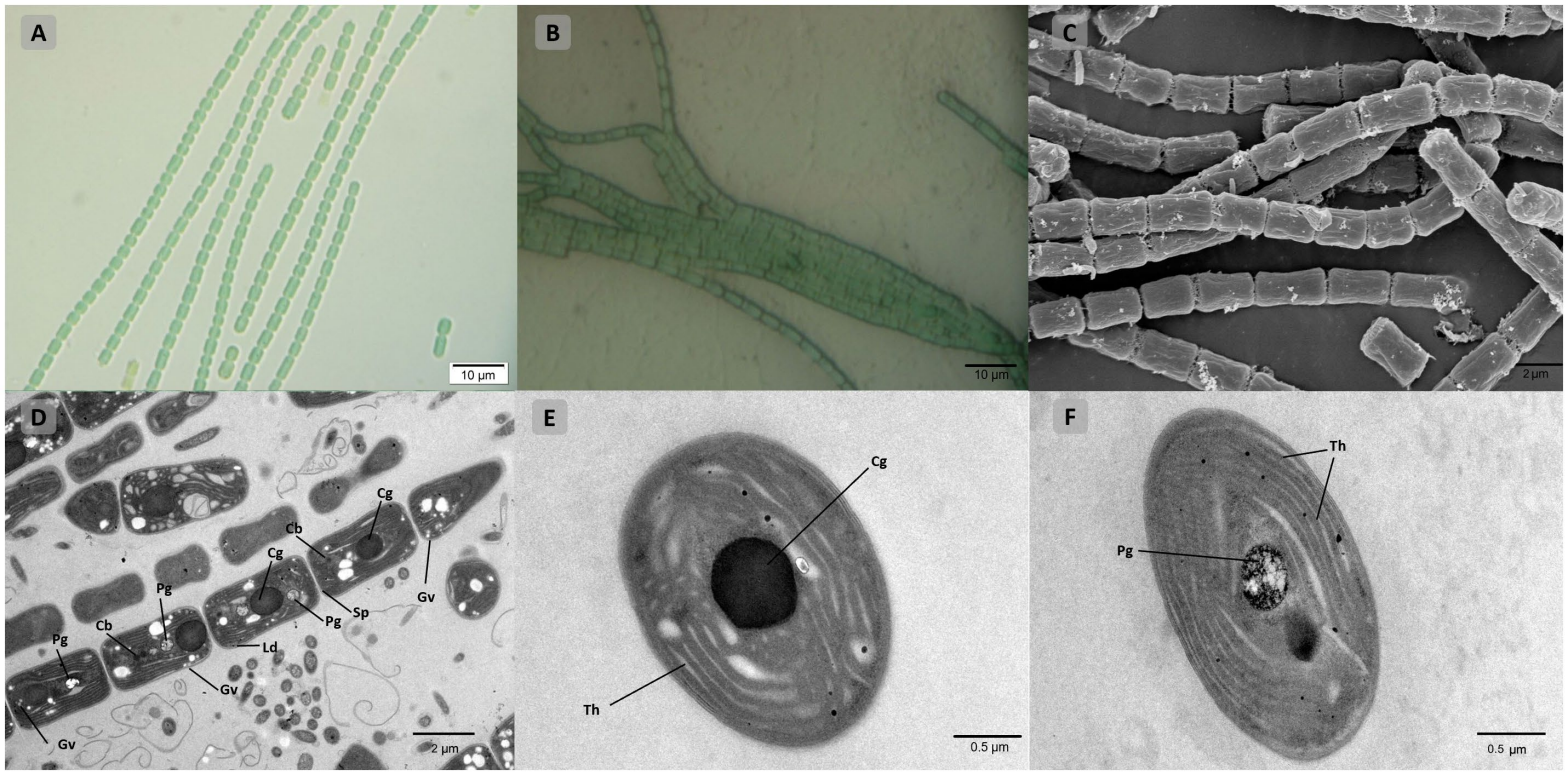
Microscopic morphology of *Trichothermofontia* B231. **(A)** Light microscopy image of liquid-medium grown cultures; **(B)** light microscopy image of solid-medium grown cultures; **(C)** SEM image; **(D–F)** TEM images. Cb, carboxysome; Cg, cyanophycin granule; Ld, lipid droplets; Pg, polyphosphate granule; Sp, septum; Th, thylakoid membrane, Gv, gas vesicles. Magnifications were 1,000× **(A,B)**; 5,000× **(C,D)**; 20,000× **(E,F)**.

Light, scanning, and transmission microscopy were utilized for the morphological description and delineation of strain B231 as the representative of a new genus and to illustrate its key characteristics. The morphology of the strain is presented in Figure 7. The unicyanobacterial culture consists of blue-green filaments (Figures 7A,B), sometimes forming clumps or flat mats when grown in stationary liquid cultures. Trichomes of the cells grown in the liquid medium are solitary or in colony-like mats, straight or bent, moderately entangled or curved, and contain elongated cylindrical barrel-shaped cells terminated with mostly sharply pointed, conical apical cells (Figures 7A,C). Cells grown on a solid medium had multiple trichomes in one sheath (Figure 7B).

Cells of the strain were longer than they were wide (2.0–2.4 μm wide, 3.4–5.0 μm long), with a length-to-width ratio ranging from 1.4–2.0, and with an irregular ratio in apical cells. The trichomes of this strain were thinner than those of other members of *Trichocoleus* (Table 6), which typically have length-to-width below 1.0 and *Pinocchia* with very diverse measurements for both cell lengths and widths. The sheath was not evident in liquid cultures but more apparent in solidified medium, cells were connected with hyaline bridges, slightly constricted at poorly visible cross-walls, with the content mostly homogeneous (Figures 7A,B). The number of thylakoids (Figures 7E,F) varies from 5 to 8 layers, and peripheral thylakoids were arrayed layer by layer, parallel to the long axis of the filament, occasionally with an irregular arrangement. The presence of large cyanophycin granules, often in the center of the cell, characterized the cytoplasm (Figures 7D,E). The relevant biosynthetic genes encoding cyanophycin synthase (*cphA*) and cyanophycinase (*cphB*) were identified in the genome. The carboxysomes were observed in the cytoplasm in small numbers, similar to related *Pinocchia* strains (Dvorak et al., 2015; Kim et al., 2021). Gas vesicles and polyphosphate granules were also observed in the cytoplasm (Figure 7D). Gas vesicles were composed of cylindrical structures, typically two proteins GvpA forming the vesicle core and GvpC providing structural support and were responsible for the shape of gas vesicles (Miklaszewska et al., 2012). Analysis of the B231 genome confirms the presence of the *gvpA*, *gvpC* genes. The other genes involved in gas vesicle formation were also identified.

**Table 6:** Comparison of morphological features of *Trichothermofontia* and closely related strains.

Species	Morphology	Cell width (um)	Cell length (um)	Sheaths	Thylakoids No.	Color	References
*Trichothermofontia sichuanensis* B231	Straight solitary unbranched filaments, multiple filaments in one sheath when grown on solid medium	2.0–2.4	2.0–5.0	Thin, colorless	5–7	Blue-green	This study
*Trichocoleus desertorum* LSB90	Straight, solitary, unbranched filaments, multiple filaments in one sheath	3.9 *±* 0.3	3.0 *±* 0.5	Thick, colorless	NA	Green	Mehda et al. (2021)
*Trichocoleus desertorum* CAU7	Straight, solitary, unbranched filaments, multiple filaments in one sheath	2.8 ± 0.4	2.0 ± 0.5	Colorless	NA	Green	Roncero-Ramos et al. (2019)
*Trichocoleus desertorum* ATA4-8-CV2	Entangled, solitary, unbranched filaments, multiple filaments in one sheath	2.5–3.8	1.5–5.5	Colorless	NA	Blue-green	Mühlsteinova et al. (2014)
*Trichocoleus abiscoensis*	Solitary, 1–4 to more trichomes in one sheath	1.0–2.0	NA	Colorless	NA	Blue-green	Komárek and Kovacik (2013)
*Trichocoleus sociatus* LSB16	Solitary, unbranched filaments, multiple filaments in one sheath	5.2 *±* 0.2	3.3 *±* 0.4	Colorless	NA	Green	Mehda et al. (2021)
*Trichocoleus sociatus* SAG 26.92	30 trichomes in one sheath	2.0–2.8	NA	Colorless	NA	Blue-green	Lange et al. (1992)
*Trichocoleus cf. delicatulus* (W. et G.S. West) Anag.	Flexuous, spreading, ropy fascicles, unbranched filaments	1.2–1.6	1.6–3.5	Colorless	NA	Olive	Flechtner et al. (2009)
*Trichocoleus* sp. 1	Slightly flexuous, tapering, unbranched filaments, 1–2 trichomes per sheath	2.5–3.0	1.5–3.5	Thin, colorless	NA	Blue-green	Alwathnani and Johansen (2011)
*Pinocchia polymorpha* E5	Straight solitary unbranched filaments	1.09–2.86	1.28–8.63	Thin, colorless	NA	Blue-green	Dvorak et al. (2015)
*Pinocchia daecheonga* FBCC-A230	Straight or bent filaments, constricted at cross-walls	1.04–1.87	1.57–5.99	Thin, colorless	NA	Blue-green	Kim et al. (2021)

NA, not available.

As shown in Table 6, the overall morphology of strain B231 was more similar to *Pinocchia* spp. (Dvorak et al., 2015; Kim et al., 2021) than to other members of the *Trichocoleusaceae* family (Lange et al., 1992; Flechtner et al., 2009; Alwathnani and Johansen, 2011; Komárek and Kovacik, 2013; Roncero-Ramos et al., 2019; Mehda et al., 2021). This difference can be linked to the habitat. The family appears to have two morphologies, probably driven by environmental conditions. The primarily terrestrial strains, such as *T. desertorum,* have a very thick sheath capable of maintaining multiple trichomes. Meanwhile, freshwater strains, such as *Pinocchia* spp. and the B231, exhibit phenotypic plasticity showing marginal sheath when grown in liquid cultures and thick sheath when grown on a solid medium.

## Conclusion

In this manuscript, the results of 16S rRNA phylogeny, secondary structures of 16S-23S ITS and strain’s morphology strongly supported the B231 as a novel genus within *Trichocoleusaceae* and its delineation as a representative of this new taxon. Phylogenomic inference and three genome-based indices (ANI, AAI, and POCP) also support the delineation at the genus level. Consequently, we have proposed a new genus *Trichothermofontia sichuanensis* gen. et sp. nov. Moreover, based on the results, we suggest family-level revision of *Pinocchia* from *Leptolyngbyaceae* to *Trichocoleusaceae*. The delineation was strongly supported at the molecular level, and its morphological distinction from *Trichocoleus* sp. can be attributed to the different habitats (terrestrial vs. freshwater). In addition, the obtained complete genome of the newly delineated *Trichothermofontia* B231 facilitates the elucidation of genetic basis regarding genes related to CCM. The strain belongs to β-cyanobacteria according to its β-carboxysome shell protein with 1B form of RubisCO. Compared to other thermophilic strains, strain B231 contains a relatively low diversity of bicarbonate transporters (only BicA for HCO_3_^−^ transport) but a higher abundance of different types of carbonic anhydrase (CA), β-CA (*ccaA*) and γ-CA (*ccmM*). Strain B231 with freshwater origin lacks the BCT1 transporter, which is consistently possessed by freshwater cyanobacteria. Furthermore, strain B231 shows a similar composition of carboxysome shell proteins (*ccmK1-4*, *ccmL*, -*M*, -*N*, -*O*, and -*P*) to mesophilic cyanobacteria, the diversity of which was higher than many thermophilic strains lacking at least one of the four *ccmK* genes. The genomic distribution of CCM-related genes suggests various regulations of expression. Overall, the first complete genome of a new genus representative obtained in this study provides insight into the genomic features, CCM components, and the fundamental information for future global taxogenomic, ecogenomic and geogenomic studies.

### Taxonomic treatment and description of *Trichothermofontia sichuanensis* Daroch, Tang, and Zhou et al. gen. et sp. nov.

**Phylum**: Cyanobacteria

**Order**: Synechococcales

**Family**: *Trichocoleusaceae*

**Genus**: Trichothermofontia, gen. nov.

***Description***: Filaments solitary, entangled, lacking false branching, forming small or short fragments in a nutritional deficiency state. Sheath colorless, thin. Trichomes straight or curved, terminated with apical, rounded cells, with invisible crosswalls that separate each cell. Cells mostly longer than wide, with multilayer peripheral thylakoids.

***Etymology***: Gr. fem. n. *thrix (gen. trichos)*, hair; Gr. masc. Adj. *thermos*, hot; L. masc. n. *fons (gen. fontis)*. “*Tricho*” referring to Greek word for hair, representing exhibiting typical thin morphology of trichome, “thermo” referring to Greek word for heat and representing thermophilic (high temperature tolerant) character of the strain, “fontia”—a genus epithet derived from the Latin word *fons* meaning spring, since all representative of the genus to date were hot spring isolates.

***Type species***: *Trichothermofontia sichuanensis* Daroch, Tang, and Zhou et al. sp. nov.

### *Trichothermofontia sichuanensis* Daroch, Tang, and Zhou et al. gen. et. sp. nov.

***Diagnosis***: Differing from other species of the genus based on the 16S rRNA sequence identity.

***Description*** Colony bright green, flat and tightly packed in a recognizable interleaved or tangled reticular morphology on agar plates. Filaments long or short, blue-green, no branching, typically solitary straight or bent, moderately entangled or curved (Figures 7A,B). Trichomes contained elongated cylindrical barrel-shaped cells terminated with conical, sharply pointed apical cells, mat-forming in stationary liquid culture, yielding solitary filaments when shaken. Cells rectangular in cross-section, connected with hyaline bridges. Intracellular connections between vegetative cells not present. Cells typically longer (3.4–4.0 μm), than wide (2.0–2.4 μm), with a length-to-width ratio ranging from 1.4 to 2.0 (Figures 7A–D), with peripheral thylakoids arrayed in 5 to 8 layers (Figures 7E,F). The sheath minimal in liquid cultures (Figure 7A), and visible in solid-medium grown cultures (Figure 7B). Carboxysomes observed in small numbers. Large centrally positioned cyanophycin granule (Figures 7D,E), and membrane-delimited vesicles (polyphosphate granules and gas vesicles) in the cytoplasm. Development of heterocytes not observed, genetic toolkit for molecular nitrogen fixation detected.

***Etymology:*** “*sichuanensis*” species epithet derives from the name of the collection province is B231 (=FACHB-3573).

***Type locality*:** Thermal spring, Zhonggu village in Ganzi Prefecture of Sichuan Province, China.

Ecology of type locality: the sample occurred as a macroscopic dark green mat attached to the rock.

***Habitat*:** Thermal springs in Ganzi Prefecture of Sichuan Province, China (30°36′39″ N, 101°41′9″ E; Supplementary Figure 5).

***Holotype here designated***: The dried inactive holotype was deposited in the Herbarium of North Minzu University with the voucher number: NMU00231 (contact: Lei Zhang, zhangsanshi-0319@163.com).

***Reference strain***: The culture of *Trichothermofontia sichuanensis* Daroch, Tang, and Zhou et al. gen. et sp. nov. was initially denoted and deposited in Peking University Algae Collection as PKUAC-SCTB231 has also been deposited in the Freshwater Algae Culture Collection at the Institute of Hydrobiology (FACHB-collection) with accession number FACHB-3573 as *Trichocoleusaceae* sp. species after identification and authentication based on the full-length sequencing of the 16S rRNA gene along with folding of the secondary structures of the 16S–23S ITS region. After proper identification and authentication, the culture is maintained in the FACHB under the accession number FACHB-3573.

## Data availability statement

The datasets presented in this study can be found in online repositories. The names of the repository/repositories and accession number(s) can be found in the article/Supplementary material.

## Author contributions

JT: conceptualization, methodology, validation, formal analysis, investigation, data curation, writing-original draft, writing-review and editing, visualization, supervision, project administration, and funding acquisition. HZ: formal analysis, investigation, data curation, and writing-original draft. YJ: formal analysis, investigation, data curation, and writing-review and editing. DY: formal analysis, investigation, and data curation. KW: methodology, validation, data curation, and writing-review and editing. L-MD: methodology, software, and data curation. MD: conceptualization, methodology, resources, data curation, writing-original draft, writing-review and editing, supervision, project administration, and funding acquisition. All authors contributed to the article and approved the submitted version.

## Funding

This research was funded by the National Natural Science Foundation of China (31970092, 32071480, and 3221101094) and Tenure-Track Fund to MD. Funding bodies had no influence over the design and execution of this research.

## Conflict of interest

The authors declare that the research was conducted in the absence of any commercial or financial relationships that could be construed as a potential conflict of interest.

## Publisher’s note

All claims expressed in this article are solely those of the authors and do not necessarily represent those of their affiliated organizations, or those of the publisher, the editors and the reviewers. Any product that may be evaluated in this article, or claim that may be made by its manufacturer, is not guaranteed or endorsed by the publisher.

## Footnotes

^1^http://enve-omics.ce.gatech.edu/g-matrix/
