# Supplementary material for: Characterization of a novel thermophilic cyanobacterium within Trichocoleusaceae, Trichothermofontia sichuanensis gen. et sp. nov., and its CO2-concentrating mechanism
Source: Front Microbiol. 2023 Apr 27;14:1111809. doi: 10.3389/fmicb.2023.1111809 (PMC10172474; doi:10.3389/fmicb.2023.1111809)
Supplement: Supplementary file 2 [file Data_Sheet_1.DOCX]

**
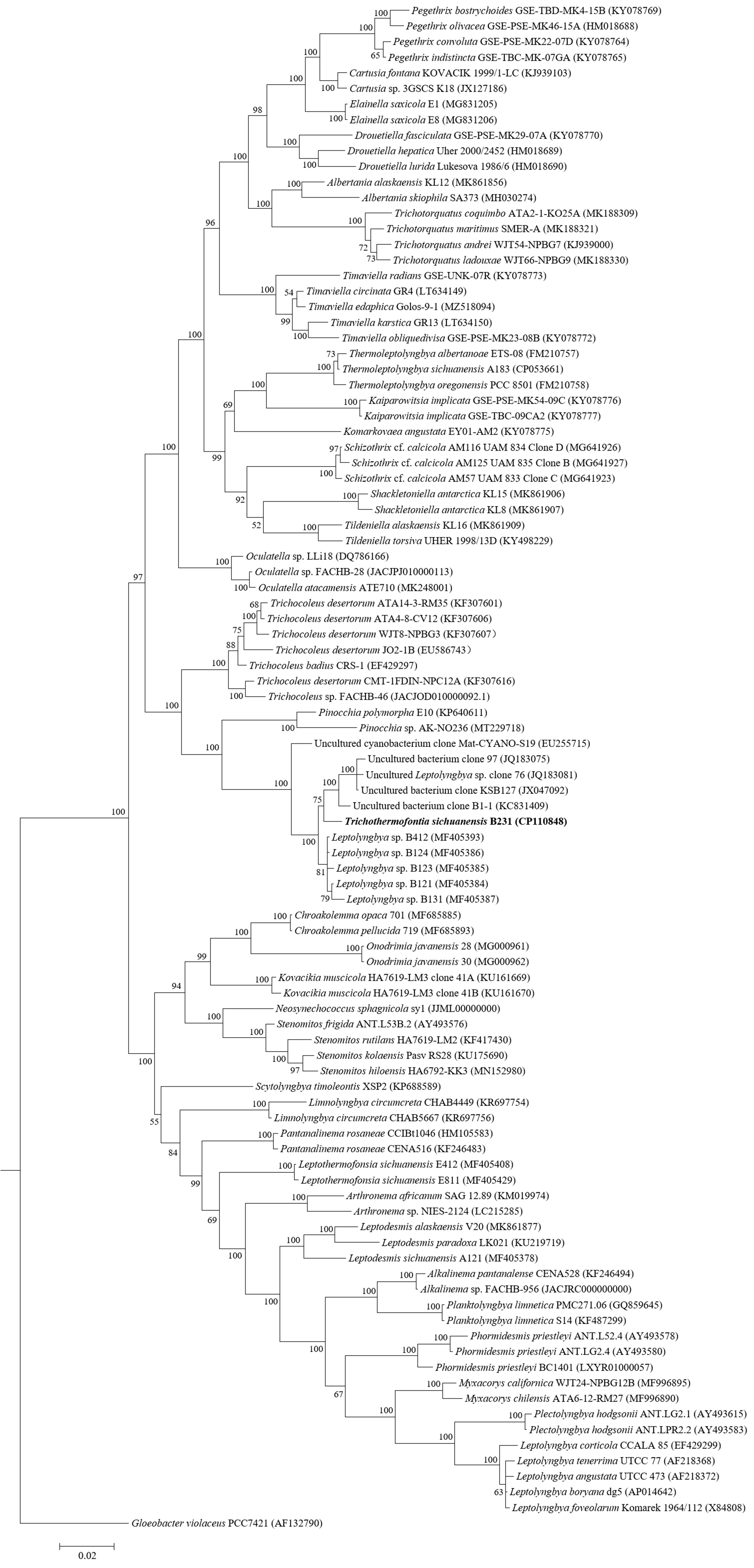
**

**Supplementary Figure 1** Bayesian inference of 16S rRNA gene sequences representing 98 cyanobacterial strains. Posterior probabilities (%) are given above the nodes.


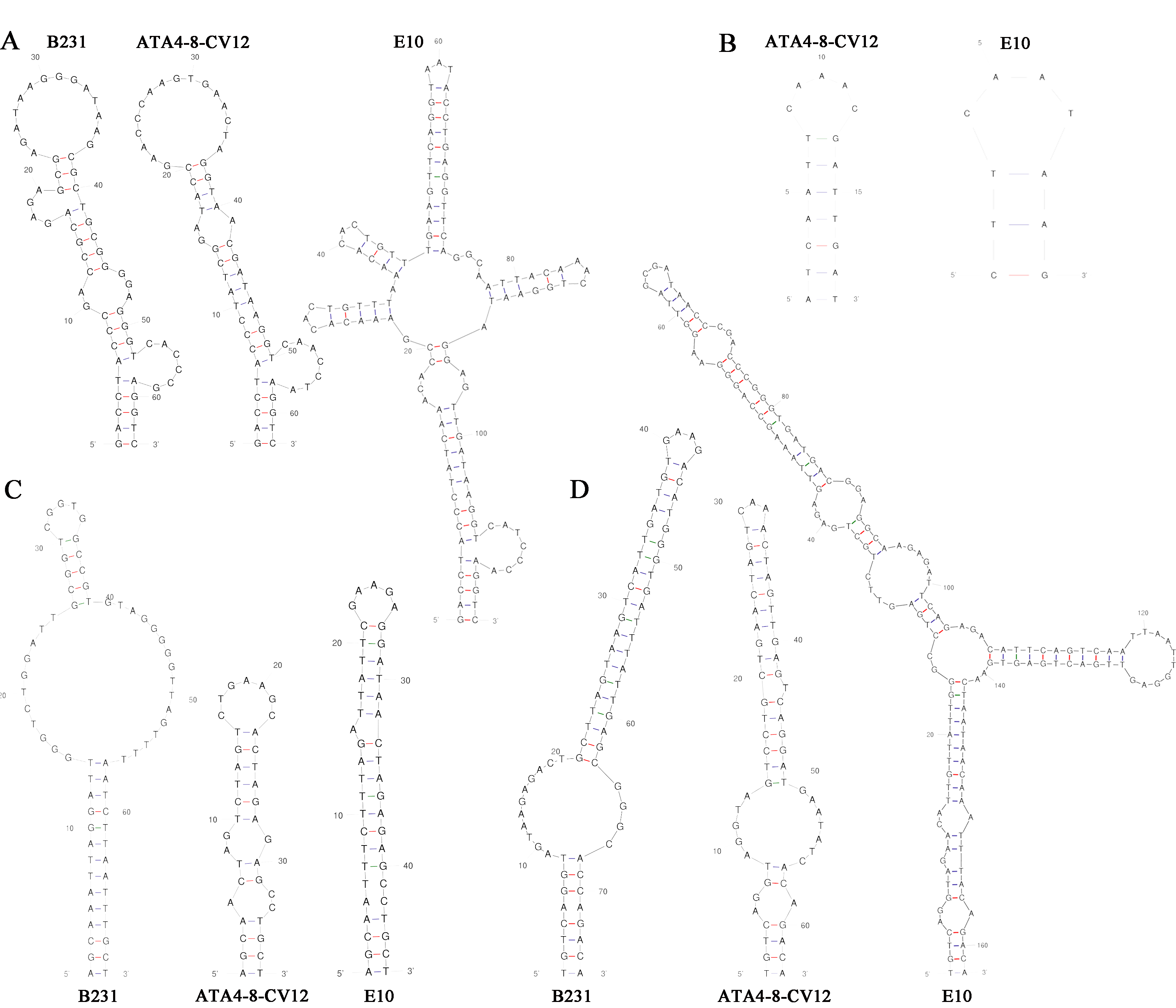


**Supplementary Figure 2** Hypothetical secondary structures of D1-D1’ helix (**A**), V2 helix (**B**), boxB (**C**) and V3 helix (**D**) of 16S-23S ITS of representative species from family Trichocoleusaceae, including *Trichothermofontia sichuanensis* B231, *Trichocoleus* *desertorum* ATA4-8-CV12 and *Pinocchia* *polymorpha* E10.


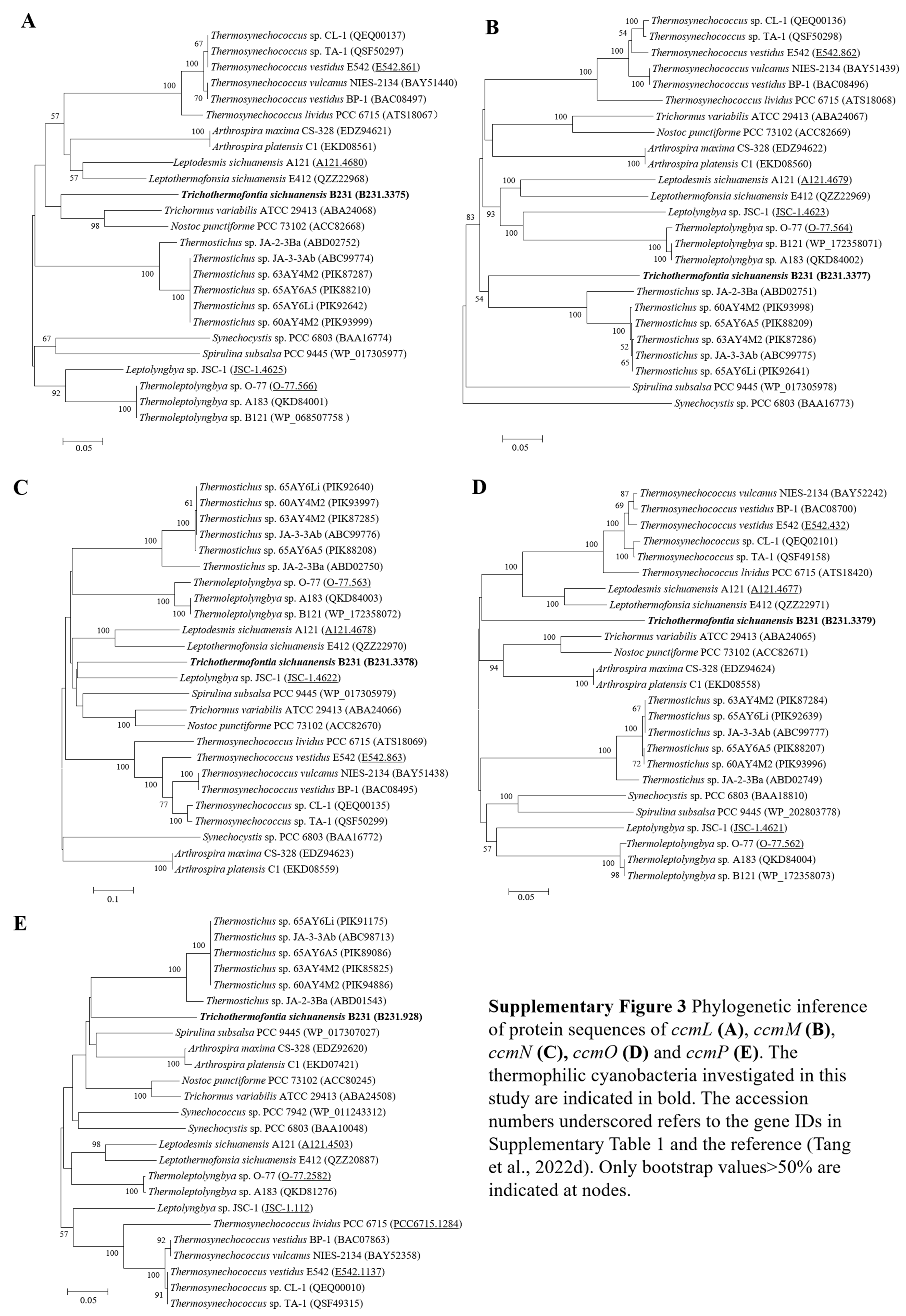


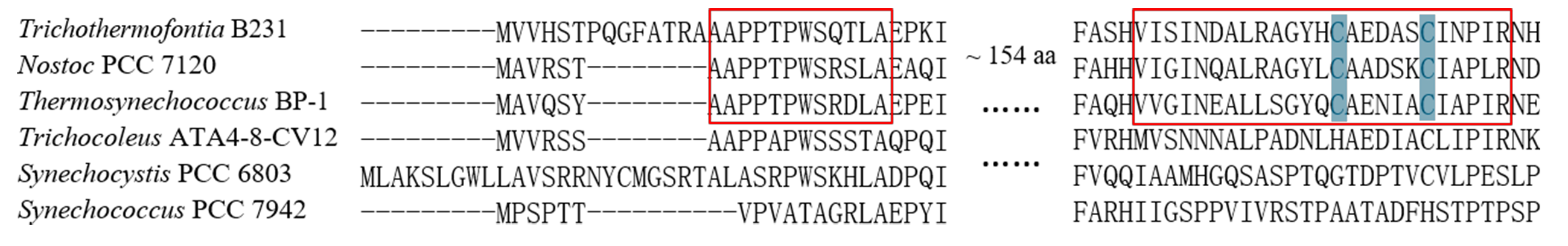


**Supplementary Figure 4** Partial alignments of *ccmM* amino acid sequences. As suggested by Peña et al. (2010), red boxes refer to conserved regions of the N-terminal domain of ccmM necessary for CA activity, while shaded cysteine amino acids indicate essential residues participating in the disulfide bond in the C-termini of active *ccmM* protein.


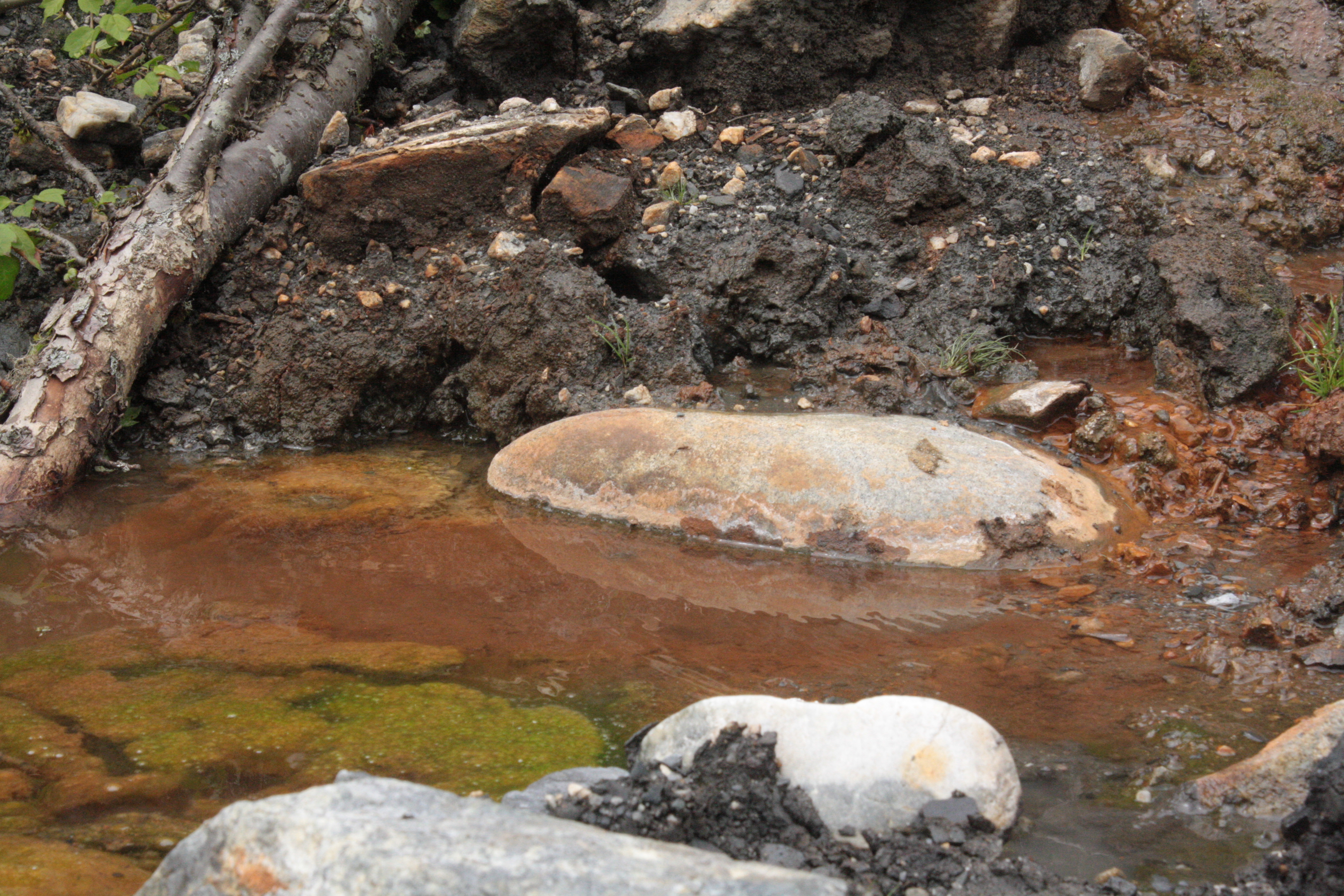


**Supplementary Figure 5** Photography of the Zhonggu village thermal spring B2 in Ganzi Prefecture of Sichuan Province, China (30°36’39” N, 101°41’9” E) from where the *Trichothermofontia sichuanensis* B231 strain was isolated.
